# Epithelial LTβR signaling controls the population size of the progenitors of medullary thymic epithelial cells in neonatal mice

**DOI:** 10.1038/srep44481

**Published:** 2017-03-14

**Authors:** Weiwei Wu, Yaoyao Shi, Huan Xia, Qian Chai, Caiwei Jin, Boyang Ren, Mingzhao Zhu

**Affiliations:** 1 Key Laboratory of Infection and Immunity, Institute of Biophysics, Chinese Academy of Sciences, Beijing, 100101, China; 2 College of Life Sciences, University of Chinese Academy of Sciences, Beijing, 100049, China

## Abstract

The establishment of T cell central tolerance critically relies on the development and maintenance of the medullary thymic epithelial cells (mTECs). Disrupted signaling of lymphotoxin beta receptor (LTβR) results in dramatically reduced mTEC population. However, whether LTβR directly or indirectly control mTECs remains undetermined; how LTβR controls this process also remain unclear. In this study, by utilizing K14-Cre × *Ltbr*^fl/fl^ conditional knockout (cKO) mice, we show that epithelial intrinsic LTβR was essential for the mTEC development postnatally. Mechanistically, LTβR did not directly impact the proliferation or survival of mTECs; the maturation of mTECs from MHC-II^lo^ to MHC-II^hi^ stage was also unaltered in the absence of LTβR; interestingly, the number of mTEC progenitors (Cld3,4^hi^SSEA-1^+^) was found significantly reduced in LTβR cKO mice at the neonatal stage, but not at E18.5. Consequently, epithelial deficiency of LTβR resulted in significant defect of thymic negative selection as demonstrated using OT-I and RIP-OVA transgenic mouse system. In summary, our study clarifies the epithelial intrinsic role of LTβR on mTEC development and function; more importantly, it reveals a previously unrecognized function of LTβR on the control of the size of mTEC progenitor population.

One of the central goals of T cell development is to achieve self-tolerance. Thymic medulla provides a unique microenvironment for this purpose. After positive selection in the thymic cortex, semimature thymocytes are recruited into the medulla for further selection by self-peptides. Medullary thymic epithelial cells (mTECs) are not only the organizer cells for proper formation of thymic medulla, they help to recruit the positively selected thymocytes via producing chemokines such as CCL19 and CCL21^1^,^2^,^3^. Moreover, by promiscuously expressing a broad range of peripheral tissue restricted self-antigens (TRAs), mTECs can directly present the self-peptides by themselves or pass the antigens onto dendritic cells (DCs) for indirect presentation^4^,^5^,^6^,^7^. Therefore, mTECs play important roles for the establishment and maintenance of T cell central tolerance.

The development of mTECs involves multiple successive steps. mTECs, as well as cortical TECs (cTECs), are both originated from the common bipotent TEC progenitor cells from the third pharyngeal pouch during embryo organogenesis in mice^8^,^9^,^10^. Downstream of bipotent TEC progenitors, although poorly understood, current data indicate that the transitional TEC progenitor cells, known to possess cTEC traits, emerge before they are committed to mTEC or cTEC lineages^10^,^11^,^12^,^13^,^14^. Identification of cTEC and mTEC restricted progenitor cells and their regulation become an interesting topic in the field^8^,^11^,^15^,^16^,^17^,^18^. As to the identification of mTECp (medullary thymic epithelial cell progenitor), several studies have confirmed the existence. At E13.5 of mice, TECs with high expression of claudin-3 and claudin-4 (Cld3,4^hi^) were found to contain committed mTEC progenitors^16^. Recently, SSEA-1^+^ fraction within the Cld3,4^hi^ TEC population was found to maintain much higher capability of mTEC regeneration than SSEA-1^−^ fraction^17^. mTEC committed progenitors are considered to differentiate into MHC-II^lo^ mTECs and MHC-II^hi^ mTECs. In the latter population, some mTECs further mature to express Aire (autoimmune regulator), a critical factor controlling the expression of TRAs for central tolerance induction^4^,^19^. Aire^+^ mTECs are not the end stage of mTEC differentiation. With cell fate mapping approaches, terminal mTEC differentiation was found beyond the Aire^+^ stage as marked by involucrin expression^20^,^21^. Thus, proper mTEC development is determined at multiple steps. Further understanding how these different differentiation steps are regulated for the development of mTEC compartment has been an attractive topic in the field.

A set of molecules, especially some from TNF receptor family and their downstream signaling molecules, including LTβR, RANK (receptor activator of nuclear factor κ b), CD40, Traf6 (TNF receptor-associated factor 6), Nik (NFκB inducing kinase) and RelB, have been well documented for their important roles in the development of mTECs^3^,^22^,^23^,^24^,^25^,^26^. Detailed analysis of the function of these molecules would help to better understand how the complicated process of mTEC development is tightly regulated. LTβR signaling pathway is broadly involved in the development of various secondary lymphoid tissues^27^,^28^. For the thymus, ablation of LTβR signaling pathway has been reported to resulted in dramatically reduced mTEC population in both embryonic and adult animals^25^,^26^,^29^. More specifically, LTβR signaling has been found preferentially important for the development of CCL21^+^Aire^−^ mTECs, a minor population (20–30%) in postnatal thymus^30^. In addition, the terminal differentiation of Aire^+^ mTEC is also regulated by LTβR signaling delivered from positively selected thymocytes^31^. However, it is still unclear precisely how LTβR deficiency leads to reduction of mTEC compartment; whether LTβR is involved in the regulation of mTEC progenitors remains intriguing.

LTβR is broadly expressed on different types of stromal cells in the thymus, including epithelial cells, mesenchymal cells and endothelial cells. Thymic mesenchymal cells are indispensable for thymic epithelial cell development. In fact, they are the major producer of FGF7 (Fibroblast growth factor 7) and FGF10, which induce thymic epithelial cell proliferation through FGFR2-IIIb^32^,^33^,^34^. For mTECs, mesenchyme derived fibroblasts have been recently found to play an important role for their maintenance and regeneration^35^. Therefore, it remains enigmatic whether LTβR signaling directly or indirectly controls mTEC development. To specifically evaluate the role of epithelial LTβR on mTEC development, in this study, we generated *Ltbr*^fl/fl^K14^Cre^ mice, in which LTβR is specifically deleted from epithelial cells in the thymus. Our results showed that these mice largely recapitulated the mTEC defect in germ line LTβR knockout mice. Mechanistically, no obvious defect of mTECs in terms of their proliferation, apoptosis and MHC-II^lo^ to MHC-II^hi^ maturation was found in the deficiency of epithelial LTβR. Interestingly, the mTEC progenitor population, as defined by Cld3,4^hi^SSEA-1^+^, was found significantly reduced in the *Ltbr*^fl/fl^K14^Cre^ neonatal mice. However, the mTEC progenitor population was not affected in the embryonic stage in the deficiency of LTβR. Together, our study not only clarifies the epithelial intrinsic role of LTβR on mTEC development, but also reveals an unrecognized mechanism for LTβR to control mTEC progenitor population postnatally.

## Results

### Epithelial deficiency of LTβR results in decreased mTEC population postnatally

**Figure 1:**
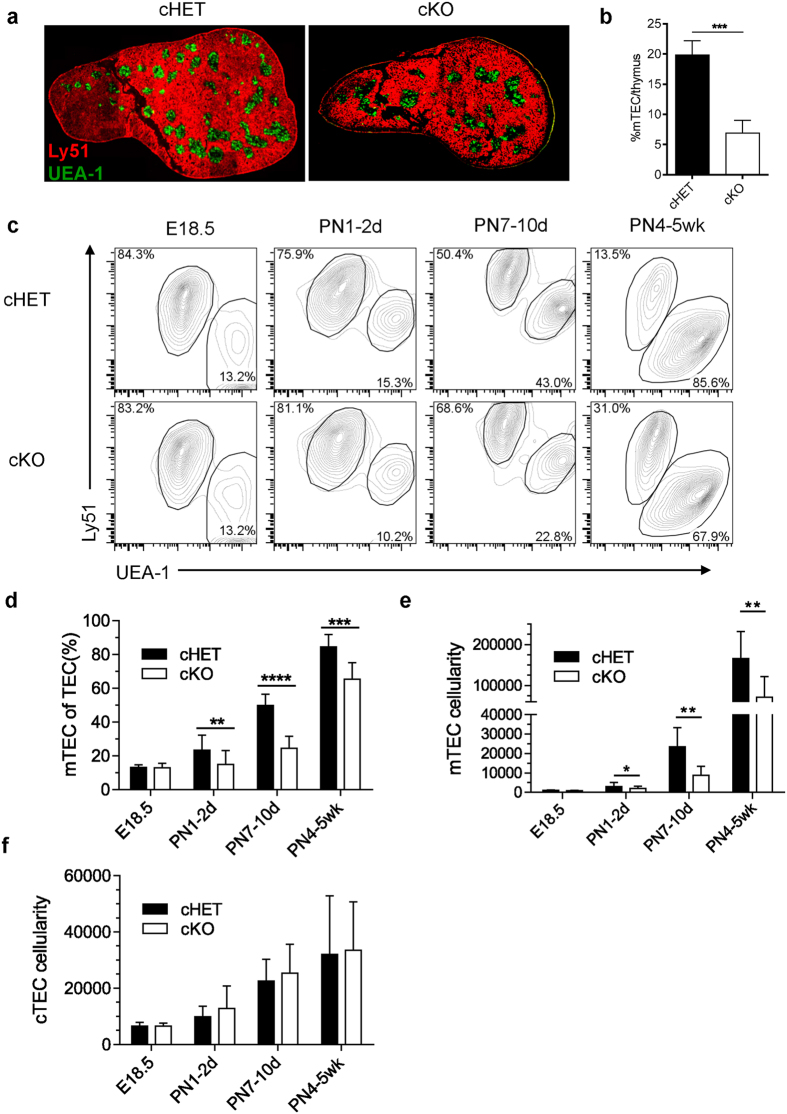
Epithelial LTβR is required for postnatal mTEC development. (**a**) The distribution of mTECs and cTECs was assessed by immunofluorescence staining of UEA-1 (green) and Ly51 (red) on the thymi from *Ltbr*^fl/fl^K14^Cre^ and *Ltbr*^fl/+^K14^Cre^ mice of 4 weeks old. The scale bar represents 1000 μm. (**b**) Quantitative analysis of thymic medulla area as determined by UEA-1 staining. Values indicate mean ± SD from at least 3 thymus sections from mice of 4 weeks old. Representative of at least 3 independent experiments. (**c**) Thymic stromal cells from *Ltbr*^fl/fl^K14^Cre^ and control mice of different ages as indicated were prepared and then stained with anti-CD45, anti-EpCAM, UEA-1 and anti-Ly51. Within TEC population (CD45^−^EpCAM^+^), mTECs and cTECs were identified as UEA-1^+^Ly51^−^ and UEA-1^−^Ly51^+^, respectively. (**d**,**e**) The frequency and absolute number of mTECs are shown. (**f**) The absolute number of cTECs is shown. One representative data was shown as mean ± SD for more than 3 mice each group. All the experiments were repeated more than three times. An unpaired two-tailed Student’s *t*-test is used: *P < 0.05; **P < 0.01, ***P < 0.001, ****P < 0.0001. cKO, conditional knockout; cTEC, cortical thymic epithelial cell; mTEC, medullary thymic epithelial cell; mTECp, medullary thymic epithelial cell progenitor; PN, postnatal.

LTβR is broadly expressed in different types of cells in the thymus. To study whether LTβR directly regulates epithelial cells for mTEC development, we generated *Ltbr*^fl/fl^K14^Cre^ mice for specific deletion of LTβR in thymic epithelial cells. *Ltbr*^fl/fl^K14^Cre^ mice demonstrated efficient TEC-specific deletion in adult mice (4–6 wks), while the deletion is less efficient at the neonatal or embryonic stages (Supplementary Figure 1). Therefore, we first analyzed the mTEC population in adult mice. The thymi develop grossly normal in *Ltbr*^fl/fl^K14^Cre^ mice. Immunofluorescence staining demonstrated obvious reduction of the thymic medulla area and loose organization of medullary islets (Fig. 1a), both of which were similarly found in germline LTβR knockout mice as reported^3^,^29^. The total medulla area is also significantly reduced in *Ltbr*^fl/fl^K14^Cre^ mice (Fig. 1b). To further quantitate the defect of mTECs, thymic epithelial cells were isolated from mice at different developmental stages for flow cytometry analysis. Dramatically reduced percentage and number of mTECs were found in adult *Ltbr*^fl/fl^K14^Cre^ mice compared with the *Ltbr*^fl/+^K14^Cre^ control mice (Fig. 1c–e). No dosage effect of LTβR was found since *Ltbr*^fl/+^K14^Cre^ mice harbored comparable frequency and number of mTEC population as in *Ltbr*^+/+^K14^Cre^ mice (Supplementary Figure 2). The cTEC population remained largely normal (Fig. 1f). Significant reduction of mTEC population was also found in *Ltbr*^fl/fl^K14^Cre^ mice at earlier postnatal ages, although at reduced degrees (Fig. 1c–e). Interestingly, however, the mTEC population at E18.5 was normal in *Ltbr*^fl/fl^K14^Cre^ mice (Fig. 1c–e). This may not be due to the inefficient LTβR deletion at earlier stages since comparable mTEC population was also confirmed in germline *Ltbr*^−/−^ mice (Supplementary Figure 3a,b). Since the K14 promoter driven Cre is active starting from the stage of bipotent TEC progenitor cells^36^, therefore deletes LTβR in both TEC lineages (Supplementary Figure 1), these results indicate a specific regulation of LTβR on mTEC development but not cTEC development at postnatal stage.

### Epithelial LTβR is not required for MHC-II and Aire expression on mTECs

**Figure 2:**
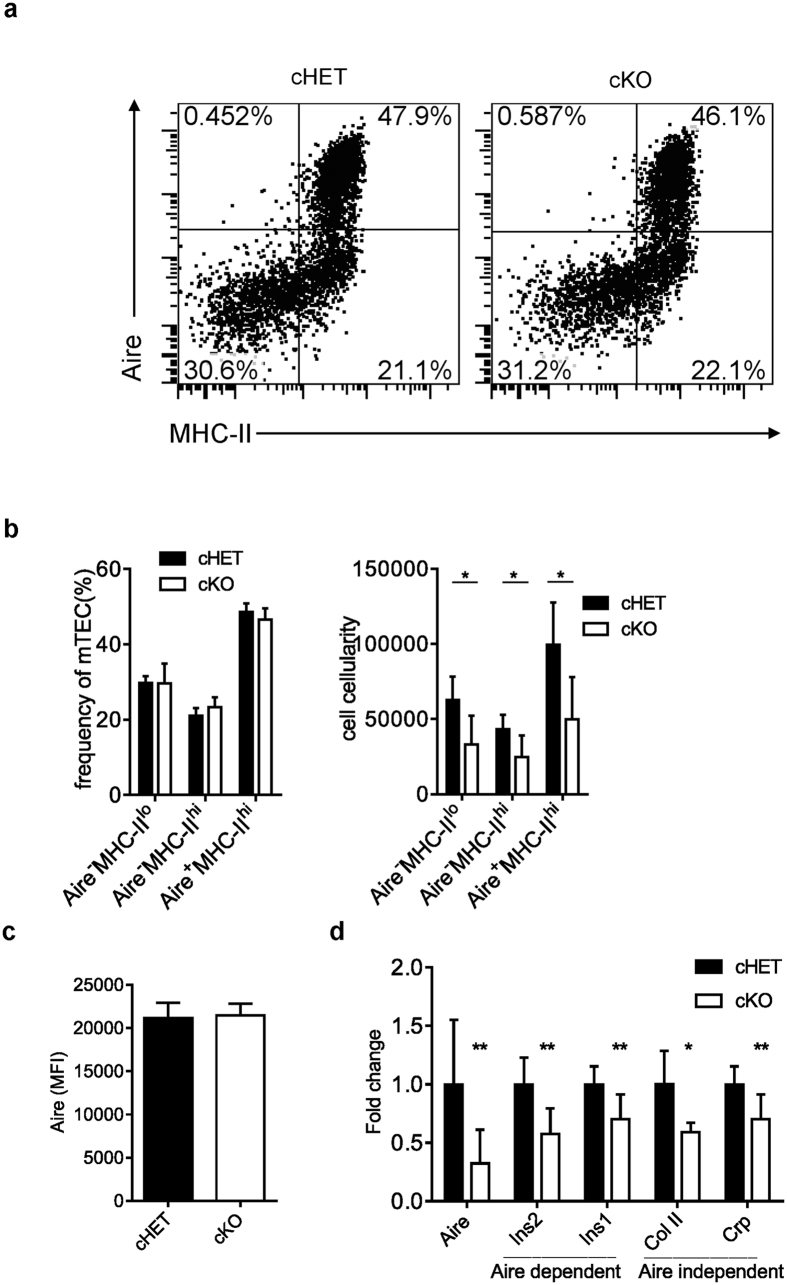
Epithelial LTβR is not required for MHC-II and Aire expression on mTECs. (**a**) After gating mTECs (CD45^−^EpCAM^+^UEA-1^+^Ly51^−^) from TECs, the maturation of mTECs in adult mice was assayed by MHC-II and Aire expression. (**b**) The frequency of each mTEC subset (Aire^−^MHC-II^+^,Aire^−^MHC-II^++^,Aire^+^MHC-II^++^) of mTECs and their absolutely numbers are shown. (**c**) MFI of Aire expression on Aire^+^MHCII^++^ mTEC subpopulation was determined by flow cytometry. (**d**) The relative mRNA expression of Aire, Ins2, Ins1, Col II and Crp in total thyme of indicated mice were determined by RT-PCR. All the experiments shown have been repeated for more than 3 times with at least 3 mice each group. An unpaired two-tailed Student’s *t*-test is used: *P < 0.05, **P < 0.01. Aire, autoimmune regulator.

Depending on the expression level of MHC-II and Aire, mTEC population during their maturation can be separated into three different subsets, MHC-II^lo^Aire^−^, MHC-II^hi^Aire^−^, MHC-II^hi^Aire^+^, among which the last subset represents the most functional mTECs for TRA expression and induction of negative selection^4^. Flow cytometry analysis demonstrated no change of the percentages of these three major subsets of mTECs in *Ltbr*^fl/fl^K14^Cre^ mice, although the numbers of these subsets were all reduced compared to those in control mice (Fig. 2a,b). In addition, the expression level of Aire was also comparable between *Ltbr*^fl/fl^K14^Cre^ mice and their littermate control mice (Fig. 2c). Thus, LTβR on epithelial cells does not seem to regulate general mTEC maturation and Aire expression on a per cell basis. Even so, the expression of Aire and both Aire-dependent TRAs (Insulin 1 and Insulin 2) and -independent TRAs (Collagen II and C-reactive protein) in total thymi were all significantly reduced in *Ltbr*^fl/fl^K14^Cre^ mice compared with those in control mice (Fig. 2d). Since the expression of none of them is reduced in sorted mTECs in *Ltbr*^fl/fl^K14^Cre^ (data not shown), this is most likely indirect due to the reduced mTEC population.

### Epithelial LTβR is not required for proliferation and apoptosis of mTECs

**Figure 3:**
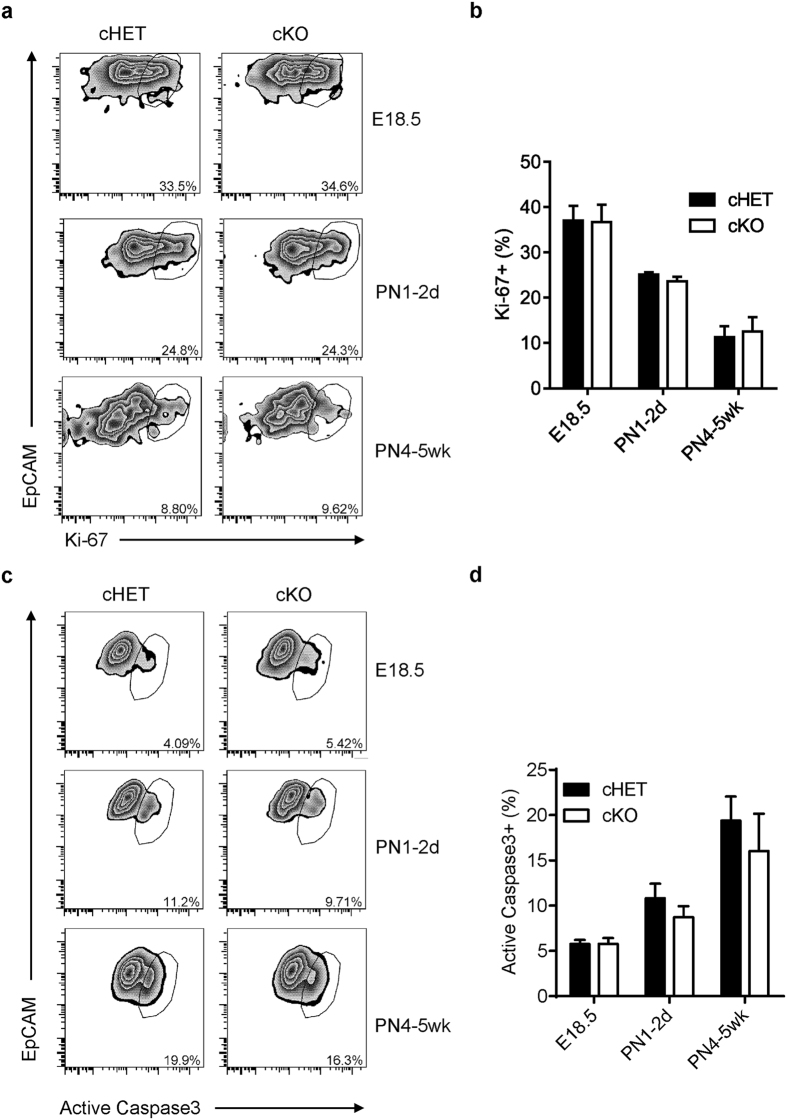
Epithelial LTβR is not required for mTEC proliferation and apoptosis. (**a**,**b**) After the surface staining, mTECs (CD45^−^EpCAM^+^UEA-1^+^Ly51^−^) from each group were intracellularly stained with Ki-67 for comparing their proliferation ability. Representative plots from mice at various time points are shown (**a**). The percentages of Ki-67 positive cells in mTECs of each group are plotted (**b**). (**c** and **d**) Intracellular staining of active caspase 3 in mTECs was used to distinguish the apoptosis cells in *Ltbr*^fl/fl^K14^Cre^ and *Ltbr*^fl/+^K14^Cre^ control mice. Representative plots from mice at various time points are shown (**c**). The percentages of active caspase 3 positive cells in mTECs of each group are plotted (**d**). All experiments have been repeated for more than 3 times with at least 3 mice per group each time. An unpaired two-tailed Student’s *t*-test is used. No significant difference was found between comparing groups.

Given the dramatic reduction of total mTEC population in *Ltbr*^fl/fl^K14^Cre^ mice, we suspected that LTβR may regulate the whole mTEC population in a more general manner. We first wondered whether the reduced mTEC population may be due to an impaired proliferation. To this end, Ki-67 was determined by flow cytometry analysis. Results showed quite normal proliferation status of mTECs in *Ltbr*^fl/fl^K14^Cre^ mice at all different developmental stages tested (Fig. 3a,b). BrdU labeling experiment further confirmed the normal mTEC proliferation in *Ltbr*^fl/fl^K14^Cre^ mice (Supplementary Figure 4). Next, we determined whether LTβR may be required for mTEC survival. To this end, the apoptotic status was measured by intracellular staining of Caspase 3 as described^37^. In fact, the percentage of apoptotic cells even had a trend to be lower in *Ltbr*^fl/fl^K14^Cre^ mice compared to the controls (Fig. 3c,d), which may reflect a compensatory feedback regulation on cell survival. Detailed analysis on MHC-II^lo^ or MHC-II^hi^ mTECs did not reveal any defects on cell proliferation or apoptosis in these subsets, either (data not shown). Thus, epithelial LTβR unlikely controls the mTEC compartment via cell proliferation/apoptosis regulation.

### Epithelial LTβR controls the number of mTECp cells

**Figure 4:**
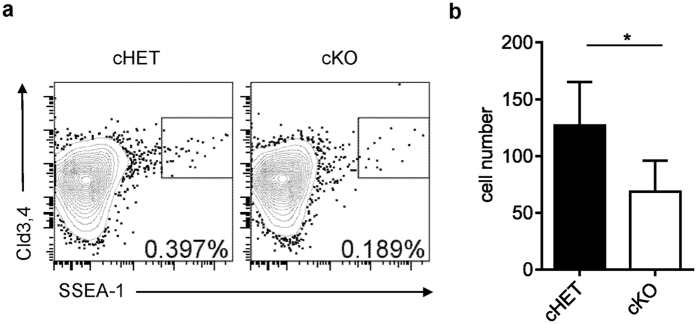
Epithelial derived LTβR controls mTECp development. (**a**) Thymic stromal cells were prepared from neonatal *Ltbr*^fl/fl^K14^Cre^ and *Ltbr*^fl/+^K14^Cre^ control mice. mTECp is identified as Cld3,4^hi^SSEA-1^+^ within CD45^−^EpCAM^+^ TEC population. (**b**) Statistic data are shown as mean ± SD of more than 3 mice each group. One representative FACS plot and statistic result from more than 3 repeats are shown. An unpaired two-tailed Student’s *t*-test is used: *P < 0.05. Cld3,4, Claudin3,4.

The largely normal mTEC proliferation and survival in the LTβR deficiency prompted to us that LTβR may not directly control the mTECp per se. We hypothesized that LTβR may function at earlier stage during mTEC differentiation. Since no cTEC defect was found in LTβR deficiency, we specifically targeted the mTEC progenitor cells. A population of Cld3,4^hi^SSEA-1^+^ thymic epithelial cells were recently found emerging at the embryonic stage, and still persist after birth^17^. This population of TECs processes much higher mTEC differentiation potential than other cells and is considered the progenitor of mTECs^17^. So far, it is still unclear how this population of cells is controlled. We checked this cell population in *Ltbr*^fl/fl^K14^Cre^ and control neonatal mice. Interestingly, a mild but consistent reduction of Cld3,4^hi^SSEA-1^+^ TECs was found in *Ltbr*^fl/fl^K14^Cre^ mice compared to the controls (Fig. 4a,b). However, the Cld3,4^hi^SSEA-1^+^ TEC population was not reduced in *Ltbr*^fl/fl^K14^Cre^ mice or straight *Ltbr*^−/−^ mice at E18.5 (data not shown and Supplementary Figure 3c,d). Together, these data suggest a novel role of LTβR in controlling the number of progenitors of mTECs, probably starting at the perinatal stage. To study the underlying mechanisms, we detected the proliferation and apoptosis of mTECp in *Ltbr*^fl/fl^K14^Cre^ and control mice. Surprisingly, however, no Ki67 staining was found in mTECp cells while non-mTECp cells are readily stained (Supplementary Figure 5a). This may be due to the slow cycling feature of stem cells or their rapid loss of the marker during their differentiation/proliferation. This may also suggest that the proliferation of mTECp per se is probably not the major contributing factor for the size of mTECp population. As to the apoptosis of mTECp cells, we did not find increased active caspase3 staining in these cells (Supplementary Figure 5b). These data suggest that LTβR may be required for the differentiation of mTECp from its progenitors (e.g. bipotent TEC progenitor cells). Given the current lack of proper tools for specific manipulation of bipotent TEC progenitor cells, this interesting hypothesis remains to be determined in future.

### Deficiency of epithelial LTβR results in impaired negative selection

**Figure 5:**
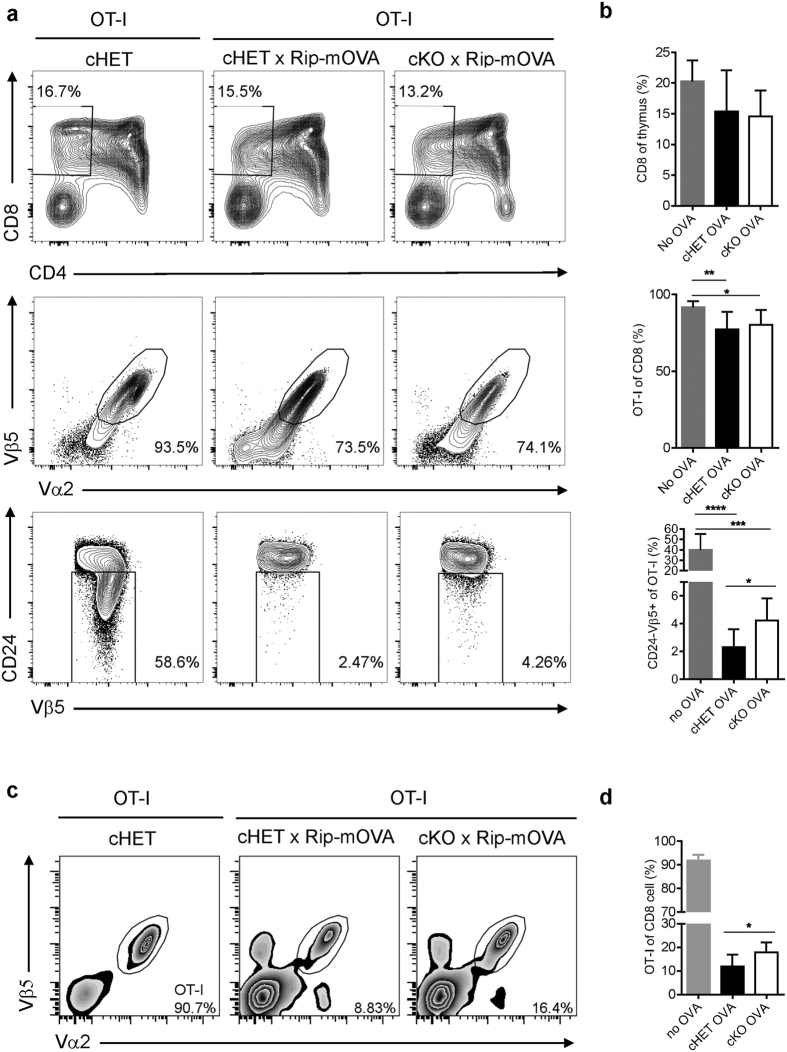
Deficiency of epithelial LTβR results in impaired negative selection. (**a**) OT-I BM cells were depleted of mature T cells and transferred into lethally irradiated recipients mice as indicated. 6 wk later, OT-I thymocyte development was analyzed by flow cytometry. Total thymocytes were first analyzed with CD4 and CD8 (*up row*) and then OT-I thymocyte gated with Vα2 and Vβ5 from CD8 single positive T cells (*middle row*). After gating OT-I thymocyte (CD4^−^CD8^+^Vα2^+^Vβ5^+^), T cell maturation was determined by CD24 (*bottom row*). Representative profiles from mice of indicated genotypes are shown. (**b**) Statistical analysis shows the pooled data from three independent experiments with 6–7 mice each group. (**c**,**d**) The proportion of splenic OT-I (CD4^−^CD8^+^Vα2^+^Vβ5^+^) was plotted (**c**) and the statistical analysis shows the pooled data from three independent experiments with 6–7 mice each group (**d**). The statistic results are shown as mean ± SD. An unpaired two-tailed Student’s *t*-test is used: *P < 0.05, **P < 0.01, ***P < 0.001, ****P < 0.0001. RIP, rat insulin promoter.

Given the significantly impaired mTEC development in *Ltbr*^fl/fl^K14^Cre^ mice, we asked further whether deficiency of epithelial LTβR alone is sufficient to lead to impaired thymoyte negative selection. To this end, we took advantage of the OT-I TCR and RIP-OVA transgenic mouse system as described before^3^. *Ltbr*^fl/fl^K14^Cre^ mice and control mice were backcrossed onto the RIP-OVA tg background. Mice were lethally irradiated and reconstituted with bone marrow cells from OT-I TCR tg mice. 6 wk later, the development of OT-I T cells were determined by flow cytometry analysis. In the OT-I bone marrow chimeric mice without OVA tg, about 93.5% CD8^+^ SP (single positive) thymocytes are Vα2^+^Vβ5^+^ and among which 58.6% are CD24^lo^ mature OT-I cells (Fig. 5a,b). In the presence of OVA tg, the total Va2^+^Vb5^+^ population and mature CD24^lo^ OT-I cells were significantly reduced, indicating efficient negative selection (Fig. 5a,b). However, epithelial deletion of LTβR resulted in significantly increased CD24^lo^ OT-I population, suggesting an escape of negative selection during OT-I thymocyte maturation (Fig. 5a,b). Consistently, the OT-I population was also significantly enriched at periphery (Fig. 5c,d). These data suggest that epithelial LTβR is required for efficient thymic negative selection.

## Discussion

Our previous study and data from others have discovered an important role of LTβR for mTEC development. However, given its broad expression, it remains unclear whether LTβR directly or indirectly controls mTEC development. In this study, we generated LTβR conditional knockout mice and tested the direct role of LTβR during mTEC development. Dramatically reduced mTEC population was found in *Ltbr*^fl/fl^K14^Cre^ adult mice, similar to that in LTβR global KO mice. This study clarified the important epithelial intrinsic function of LTβR signaling on mTEC development.

Another major finding of this study is the discovery of the regulation of Cld3,4^hi^SSEA-1^+^ mTECp population by LTβR signaling. To our knowledge, this is the first report to show a molecular control of this mTECp population size. Interestingly, however, LTβR signaling was not found essential for the mTECp at the embryonic but at the neonatal stage. Why LTβR is only required during later thymic development is intriguing. A noteworthy phenomenon of thymic development at the perinatal period is the dramatic expansion of thymocytes before E17-18^38^,^39^. Considering the important role of positively selected single positive thymocytes for mTEC development, it may suggest that these cells may be also important for mTECp population size control. In line with this, Cld3,4^hi^SSEA-1^+^ mTECp population appeared also to be reduced in size in *Rag2*^−/−^ mice, although their clonogenic function was actually increased^17^. SP thymocyte specific deletion of LT (lymphotoxin) will be needed to test this hypothesis.

The epithelial role of LTβR on mTEC development has also been studied recently using CCL19-Cre mediated LTβR deletion^40^. In this study, the authors found significant accumulation of junctional TECs (jTECs) in *Ltbr*^fl/fl^CCL19^Cre^ mice. The jTECs are proposed as mTEC committed progenitors in this study. However, in our current work, we did not find accumulation of Cld3,4^hi^SSEA1^+^ population in *Ltbr*^fl/fl^K14^Cre^ mice; but rather this population is reduced. This is not in discrepancy since different markers are used for the definition of mTEC progenitors. The relationship between these two populations in terms of their mTEC progenitor capacity is unclear. Even so, given the relatively larger size of jTEC population as compared to Cld3,4^hi^SSEA-1^+^ cells, we prefer to a hypothesis that jTEC is at the downstream step of Cld3,4^hi^SSEA-1^+^ cells. Therefore, LTβR signaling may exert different roles during early mTEC differentiation: it regulates both the size of early mTEC progenitors (Cld3,4^hi^SSEA-1^+^) and the downstream differentiation to mTECs. Both of them may contribute to the reduced mTEC compartment in *Ltbr*^fl/fl^K14^Cre^ or *Ltbr*^−/−^ mice.

Supporting this hypothesis, recently a RANK Venus reporter mouse model was created and helped to reveal an important role of RelB, a major transcription factor downstream of LTβR, in the production of RANK ^+^ mTEC progenitors^18^, which were considered to be derived from Cld3,4^hi^SSEA-1^+^ TECs. However, the Cld3,4^hi^SSEA-1^+^ progenitor cells appear not to be dependent on RelB in this study. Since we also did not detect reduced Cld3,4^hi^SSEA-1^+^ TEC population at embryonic stage in *Ltbr*^fl/fl^K14^Cre^ or *Ltbr*^−/−^ mice but at neonatal stage, one possibility may be that RelB may be involved in the generation of Cld3,4^hi^SSEA-1^+^ progenitor cells later during thymic development. This is worth to be tested in future. In addition, other possibility exists that LTβR may regulate Cld3,4^hi^SSEA-1^+^ progenitor cells independent of RelB.

In our study, the mTEC population is normal at the embryonic stage E18.5 in *Ltbr*^−/−^ mice. This is in contrast with a previous study^29^. We notice that while our *Ltbr*^−/−^ mice were originally made by Dr. Pfeffer with *neo* gene replacement of the portion between exon 1 and exon 5^41^, they generated a different line by replacing the similar region with both *LacZ* and *neo* genes. Whether this is due to the different mice used remains to be determined.

Our data showed that epithelial deficiency of LTβR leads to slightly impaired thymic negative selection. The increased population of mature OT-I thymocytes is unlikely due to the thymic emigration defect as reported in *Ltbr*^−/−^ mice^25^. Supporting this, the percentage of total CD24^lo^CD8^+^ thymocytes is not increased in *Ltbr*^fl/fl^K14^Cre^ mice (Supplementary Figure 6), suggesting a normal thymic emigration. The discrepancy on the thymic emigration defect may be due to the different mice used. The previous study used germline LTβR deficient mice, while mice with epithelial deficiency of LTβR were used in our current work. Although we also confirmed the accumulation of mature single positive thymocytes in LTβR global KO mice (data not shown), this is not the case for *Ltbr*^fl/fl^K14^Cre^ mice. This suggests that LTβR may regulate non-epithelial cells, such as endothelial cells, for thymic emigration control.

It is surprising to find that the negative selection escape in LTβR cKO thymus is not as severe as that in LTβR global KO mice as we previously reported^3^. Since mTEC population is similarly reduced in cKO as in KO mice, we consider the function of mTECs on a per cell basis is different in cKO and KO mice. Specifically for the RIP-mOVA/OT-I model, we have previously found total thymic mOVA was not reduced in the LTβR KO mice, even mTEC population was dramatically reduced, suggesting other mechanisms of LTβR controlling OT-I negative selection. Instead, the expression of CCL21 and CCL19 was significantly reduced in the mTECs of LTβR KO thymus on a per cell basis, which was further demonstrated to be a contributing reason for the escape of negative selection of OT-I cells. In our current work, we have also determined CCL21 and CCL19 expression from sorted mTECs, and found no significant reduction of CCL21 and less dramatic reduction of CCL19 (about 2-folds in cKO mice vs 8-folds in KO mice). This probably also explain why the defect of negative selection is smaller in LTβR cKO mice compared to that in LTβR total KO mice. The subdued reduction of SLC/ELC may be due to an incomplete deficiency of LTβR on mTECs. Although LTβR expression on mTECs is indeed greatly reduced in the LTβR cKO mice, about 25% residue still remains according to the MFI analysis (Supplementary Figure 1a) in our *Ltbr*^fl/fl^K14^Cre^ mice, which is consistent with a previous study^42^. Other LTβR cKO mice with possible more complete LTβR ablation, such as Foxn1-LTβR cKO, need to be tested. Other negative selection models are also worth to be tested. In addition, we cannot exclude the possibility that LTβR regulates thymic negative selection via other stromal cells.

Although epithelial deficiency of LTβR is able to break central tolerance of negative selection, we did not observe autoimmune phenotype in the old (8–10 months) *Ltbr*^fl/fl^K14^Cre^ mice as determined by lymphocyte infiltration in peripheral organs, anti-insulin antibody measurement or autoreactivity against peripheral tissues of *Rag1*^−/−^ mice. This may be partially due to the less severe defect of negative selection in *Ltbr*^fl/fl^K14^Cre^ mice compared with that in LTβR global deficient mice^3^. In addition, as mentioned above, since thymic fibroblast has also been reported to be important for mTEC development and maintenance^33^,^35^,^43^, it is possible that LTβR contribute to negative selection or other central tolerance mechanisms via fibroblasts directly or indirectly. Further investigations are required to address these issues.

In summary, our study has clarified the direct role of epithelial LTβR on mTEC development and function. Furthermore, an unexpected role of LTβR on Cld3,4^hi^SSEA-1^+^ mTECp but not mTECs per se was revealed. These data indicate the importance of mTECp for the full expansion of mTEC compartment. Targeting this mTECp population via LTβR signaling manipulation may provide novel strategies for thymic regeneration.

## Methods

### Mice

Wild type C57BL/6 mice were purchased from Vital River, a Charles River company in China. *Ltbr*^fl/fl^ and *Ltbr*^−/−^ mice were as previously described^44^,^45^, and kindly provided by Dr. Yang-Xin Fu. K14-Cre, OT-I and RIP-mOVA transgenic mice were obtained from Jackson Laboratory. For the developmental staging, the virginal plug observing day was designated as embryonic 0.5. All mice are on the C57BL/6 background and were maintained under specific pathogen-free conditions with approval by the institutional committee of Institute of Biophysics, Chinese Academy of Sciences. All animal experiments were performed in accordance with the guidelines of the Institute of Biophysics, Chinese Academy of Sciences, using protocols approved by the Institutional Laboratory Animal Care and Use Committee.

### Isolation of thymic epithelial cells

TECs were isolated largely as described previously^3^,^46^. Briefly, thymic tissues were collected, cut into 1 mm^3^ pieces and then digested with 0.5 mg/ml Collagenase I (Sigma), 1U/ml Dispase I (Corning) and 0.06 mg/ml DNase I (Roche) in 2% FBS RPMI 1640 for 5 × 20 min. The digestion was incubated with 5 mM EDTA for 5 min before washing with cold 2% FBS RPMI 1640, and filtered through a 70-μm cell strainer (Biologix Group). Stromal cells were enriched by density gradient centrifugation in Percoll (GE Healthcare). The enriched stromal cells were stained with antibodies for flow cytometry analysis.

### Immunofluorescence microscopy

Thymic tissues were embedded in OCT compound (Sakura) and snap frozen in liquid nitrogen. 8 μm cryosections were prepared, air-dried and fixed for 10 min in cold acetone. Cryosections were blocked for 1 h at room temperature in PBS containing 5% FBS and 1 mg/ml anti-FcγRII/CD16 (2.4G2) (in-house production) before staining with UEA-1-FITC (Vector Laboratories). Images were taken on a confocal microscope (Zeiss LSM-710) and analyzed with ZEN 2010 software (Carl Zeiss, Inc.) and Fiji ImageJ. To measure the surface area of thymic epithelial cells, frozen thymic sections were stained with UEA-1 and Ly51 and high resolution pictures were obtained as described above. The size of UEA-1+ area and Ly51+ area in each thymic section was quantified by ImageJ separately and statistical analysis was done by GraphPad Prism 6 with a two-tailed Student’s *t*-test.

### Bone marrow chimeric construction

*Ltbr*^fl/fl^K14^Cre^ and control mice on RIP-OVA background were irradiated with Co60 for 10 Gy. On the next day, 5 × 10^6^ OT-I bone marrow cells were intravenously transferred. Mature T cells were depleted from the bone marrow using anti-CD4 and anti-CD8 depleting antibodies. Mice were given prophylactic water containing antibiotics for 3 wk since irradiation. 8 wk after bone marrow transfer, mice were sacrificed for thymocyte and splenocyte analysis.

### Flow cytometry and antibodies

Thymic epithelial cells were acquired as described above. Cells were incubated with 2.4G2 before staining with other antibodies. The antibodies used included anti-CD45 (30-F11, eBioscience), anti-EpCAM (G8.8, Biolegend), anti-Ly51 (6C3, Biolegend), FITC labelled Ulex europaeus agglutinin-1 (UEA-1; Vector Laboratory), anti-IA/IE (M5/114.15.2, Biolegend), anti-SSEA-1 (MC-480, Biolegend), anti-LTβR (eBio3C8, eBioscience). C-CPE (C. *perfringens* enterotoxin) was produced as described^47^ and biotin-conjugated; Streptavidin APC-eFluor 780 (eBioscience) was used for visualization of C-CPE. For intracellular staining of Ki-67 (B56, BD) and active caspase 3 (C92-605, BD), cells were fixed and permeabilzated with BD Cytofix/Cytoperm™ Fixation/Permeabilization Solution Kit (554714) and stained according to the manufacture’s protocols. Fixable viability dye (L-34967, ThermoFisher) was used to exclude dead cells. For thymocyte and splenocyte analysis, cells were stained with anti-CD4 (RM4-5, eBioscience), anti-CD8 (53-6.7, eBioscience), anti-Va2 (B20.1, eBioscience), anti-Vb5 (MR9-4, eBioscience), and anti-CD24 (M1/69, Biolegend) before flow cytometry analysis. The samples were analyzed on BD LSRFortessa and FlowJo software (Tree Star Inc).

### Quantitative real-time PCR

RNA from total thymi was extracted by TRIzol (Invitrogen) according to the manufacturer’s instruction. RNA was reversely transcribed into cDNA using RevertAid First Strand cDNA Synthesis Kit (Life Technologies). Quantitative real-time PCR (qRT-PCR) was performed using SYBR Premix Ex Taq (Takara) on an ABI 7500 Real-Time PCR system. The primers used for qRT-PCR are shown as below: Aire (forward: 5′-CCAGTGAGCCCCAGGTTAAC; reverse: 5′-GACAGCCGTCACAACAGATGA-3′) Crp (forward: 5′-TACTCTGGTGCCTTCTGATCATGA-3′; reverse: 5′-GGCTTCTTTGACTCTGCTTCCA′), Coll II (forward: 5′-ATCAAACCTTTAGTGCAGAGTGG; reverse: 5′-CTGTATTCCCCGTTGTGTAGC-3′), Hprt (forward: 5′-TCCTCCTCAGACCGCTTTT; reverse: 5′-CCTGGTTCATCATCGCTAATC′), Insulin 1 (forward: 5′-CTTCAGACCTTGGCGTTGGA; reverse: 5′-ATGCTGGTGCAGCACTGATC-3′), Insulin 2 (forward: 5′-CTTCAGACCTTGGCGTTGGA; reverse: 5′-ATGCTGGTGCAGCACTGATC-3′), ΔΔCt method was used to calculate relative target gene expression to Hprt.

### Statistical analysis

All results are showed as mean ± SD. Student’s two-tailed unpaired *t*-test was used for the comparison between two groups. *P < 0.05; **P < 0.01; ***P < 0.001, ****P < 0.001.

## Additional Information

**How to cite this article:** Wu, W. *et al*. Epithelial LTβR signaling controls the population size of the progenitors of medullary thymic epithelial cells in neonatal mice. *Sci. Rep.* **7**, 44481; doi: 10.1038/srep44481 (2017).

**Publisher's note:** Springer Nature remains neutral with regard to jurisdictional claims in published maps and institutional affiliations.

## Supplementary Material

Supplementary Figures
