# Supplementary material for: Epithelial LTβR signaling controls the population size of the progenitors of medullary thymic epithelial cells in neonatal mice
Source: Sci Rep. 2017 Mar 14;7:44481. doi: 10.1038/srep44481 (PMC5349570; doi:10.1038/srep44481)
Supplement: Supplementary Figures [file srep44481-s1.pdf]

Epithelial LTβR signaling controls the population size of the progenitors of medullary thymic epithelial cells in neonatal mice

Weiwei Wu[1,2,3], Yaoyao Shi[1,2,3], Huan Xia[1,2], Qian Chai[1], Caiwei Jin[1,2], Boyang Ren[1,2] & Mingzhao Zhu[1,2]

[1] Key Laboratory of Infection and Immunity, Institute of Biophysics, Chinese Academy of Sciences, Beijing 100101, China.

[2] University of Chinese Academy of Sciences, Beijing 100049, China.

[3] W.W. and Y.S. contributed equally to this work.

Correspondence to: Mingzhao Zhu, Key Laboratory of Infection and Immunity, Institute of Biophysics, Chinese Academy of Sciences. 15 Da Tun Rd, Chaoyang District, Beijing 100101, China. Tel: 86-10-64888775; Fax: 86-10-64884618; Email: zhumz@ibp.ac.cn

Figure S1. LTβR is specifically deleted from thymic epithelial cells.

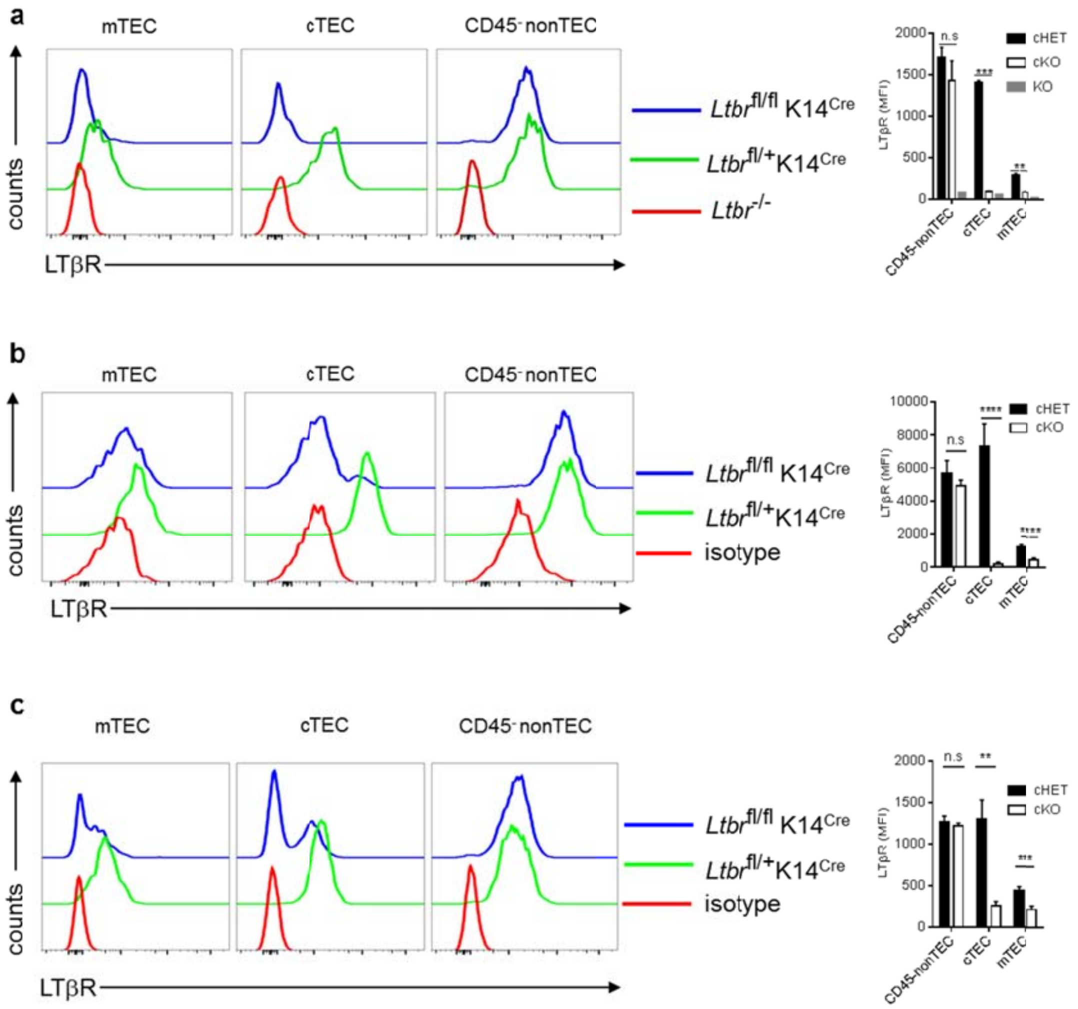

Figure S1. LTβR is specifically deleted from thymic epithelial cells. Thymic stromal cells were prepared as described in Materials and Methods. (**a**) LTβR expression was detected by flow cytometer staining on mTECs ($CD45^-$ $EpCAM^+UEA\text{-}1^+Ly51^-$), cTECs ($CD45^-EpCAM^+UEA\text{-}1^-Ly51^+$) and non-TEC stromal cells ($CD45^-EpCAM^-$) from *Ltbr*$^{-/-}$, *Ltbr*$^{fl/fl}$K14^{Cre} and *Ltbr*$^{fl/+}$ K14^{Cre} mice of 4-6 wks old. Representative FACS staining is shown on the left and the right shows the statistical analysis of MFI of LTβR expression. (**b**, **c**) Similar as described above, LTβR expression was detected by flow cytometer staining on mTECs, cTECs and non-TEC stromal cells from *Ltbr*$^{fl/fl}$K14^{Cre} and *Ltbr*$^{fl/+}$ K14^{Cre} mice of postnatal day 1 (**b**) and embryonic 15.5 (**c**). The statistical data are shown as mean ± SD for more than 3 mice each group. An unpaired two-tailed Student's *t*-test is used: n.s, no significant, **, $P<0.01$, ***, $P<0.001$, ****, $P<0.0001$. Representative data of two independent experiments.

Figure S2. LTβR has no dosage effect on the number of mTECs.

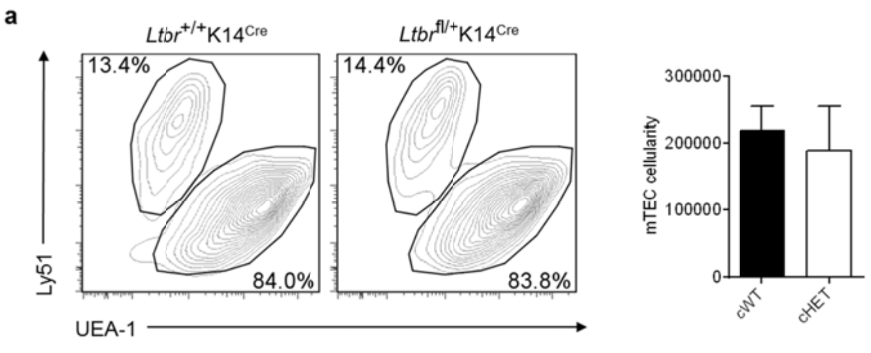

Figure S2. LTβR does not have dosage effect on the number of mTECs. (**a**) Thymic stromal cells from 4-6 wks old *Ltbr*$^{+/+}$ K14^{Cre} (WT) and *Ltbr*$^{fl/+}$K14^{Cre} mice were prepared and then stained with anti-CD45, anti-EpCAM, UEA-1 and anti-Ly51. Within TEC population (CD45^{-}EpCAM^{+}), mTECs and cTECs were identified as UEA-1^{+}Ly51^{-} and UEA-1^{-}Ly51^{+}, respectively. Representative plot is shown. (**b**) The numbers of mTECs are analyzed. Statistic data are shown as mean ± SD of 3 mice each group. An unpaired two-tailed Student's *t*-test is used. Representative data of two independent experiments.

Figure S3. Both mTEC and mTECp populations are unaltered in E18.5 *Ltbr*$^{-/-}$ mice.

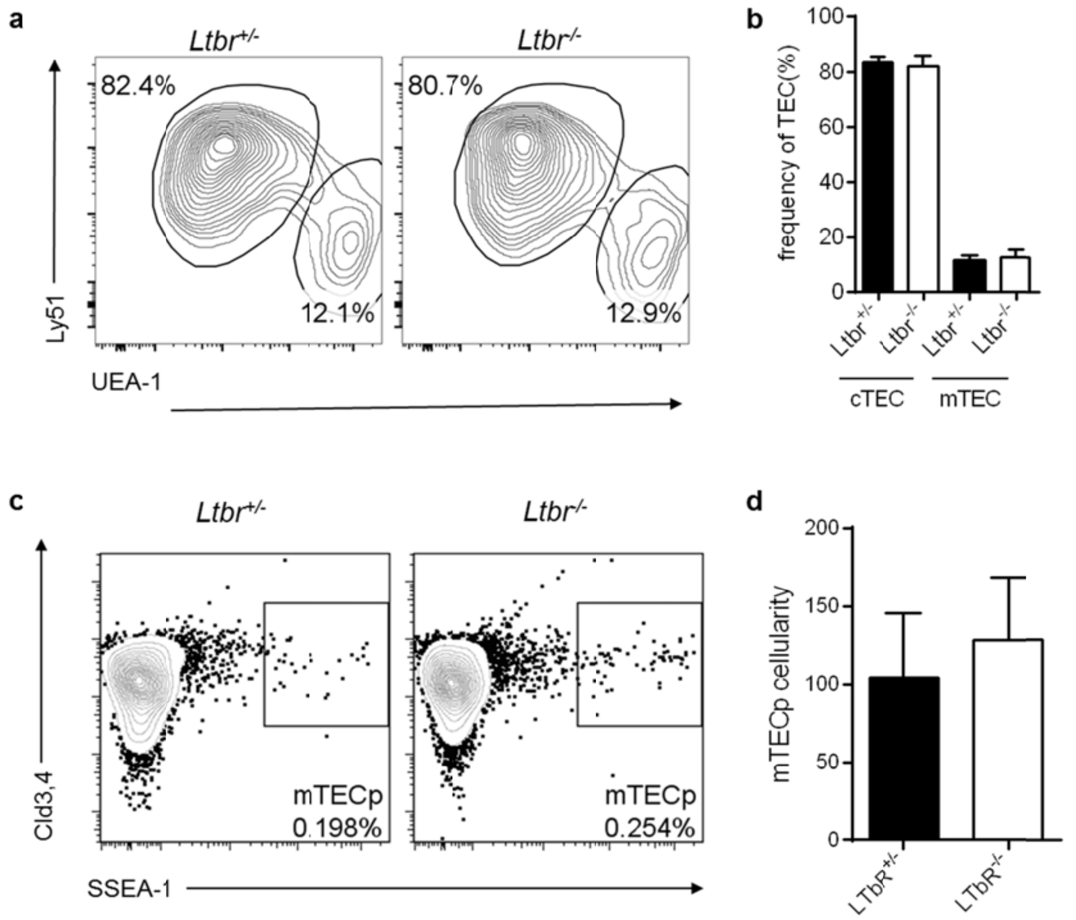

Figure S3. Both mTEC and mTECp populations are unaltered in E18.5 *Ltbr*$^{-/-}$ mice. (**a**) Thymic stromal cells from E18.5 *Ltbr*$^{-/-}$ and *Ltbr*$^{+/-}$ mice were prepared and then stained with anti-CD45, anti-EpCAM, UEA-1 and anti-Ly5**1**. Within TEC population (CD45^{-}EpCAM^{+}), mTECs and cTECs were identified as UEA-1^{+}Ly51^{-} and UEA-1^{-}Ly51^{+}, respectively. (**b**) The frequency of mTECs and cTECs are shown. (**c**) Thymic stromal cells from E18.5 *Ltbr*$^{-/-}$ and *Ltbr*$^{+/-}$ mice were prepared. mTECp is identified as Cld3,4^{hi}SSEA-1^{+} within CD45^{-}EpCAM^{+} TEC population. (**d**) The numbers of mTECp cells are analyzed. Statistic data are shown as mean ± SD of 4-5 mice each group. An unpaired two-tailed Student's *t*-test is used. Representative data of two independent experiments.

Figure S4. mTEC proliferation as determined by BrdU labeling.

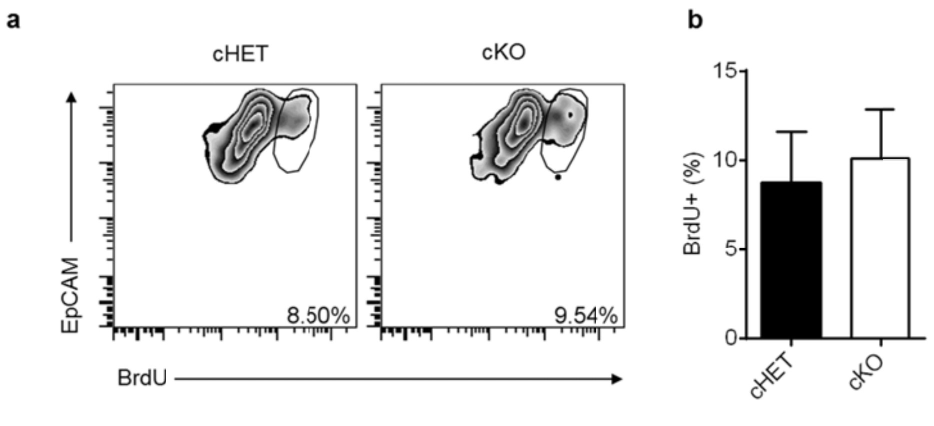

Figure S4. mTEC proliferation as determined by BrdU labeling. 4 wks old *Ltbr*$^{fl/fl}$K14^{Cre} and *Ltbr*$^{fl/+}$ K14^{Cre} control mice were treated intraperitoneally with BrdU (1mg/mouse) once a day for continuously two days. Two days later, thymic epithelial cells were acquired, stained as the manufacturer's instruction provided by BD Pharmingen. The representative plot shown was gated from mTECs, and BrdU positive cells represent those proliferating mTECs. Statistic data are shown as mean ± SD for 4-6 mice in each group. An unpaired two-tailed Student's *t*-test is used. Representative data of three independent experiments.

Figure S5. Detection of proliferation and apoptosis of mTECp.

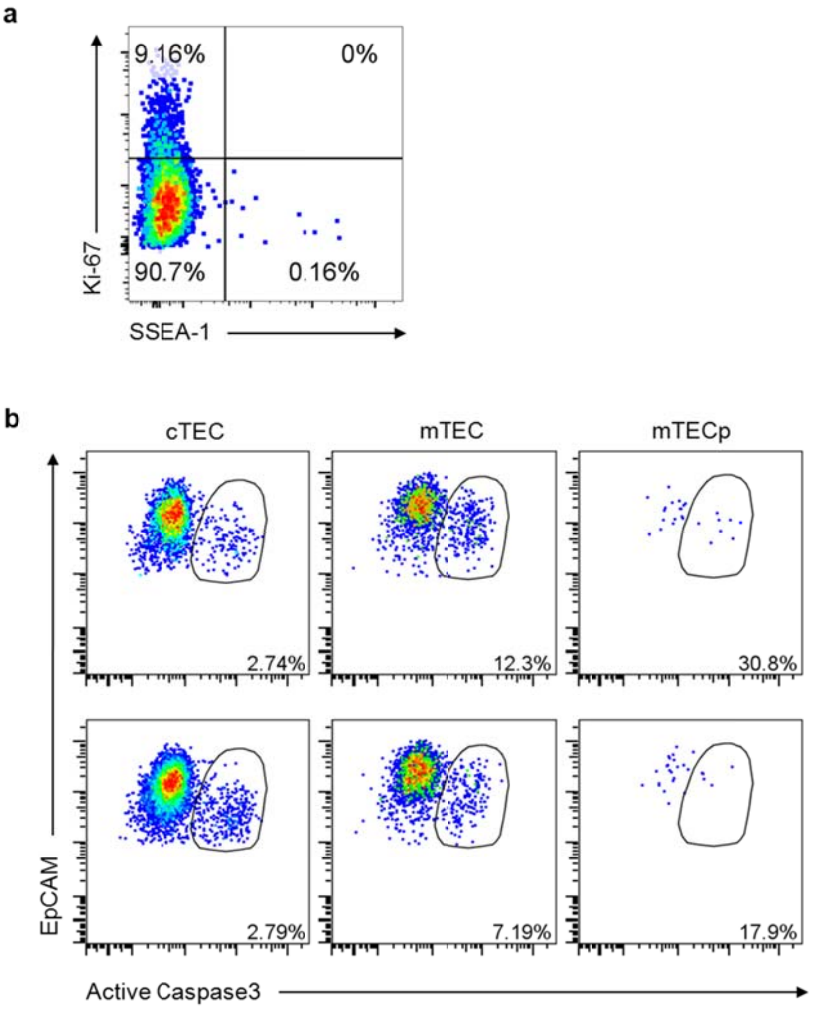

Figure S5. Detection of proliferation and apoptosis of mTECp. (**a**) Thymic stromal cells from 4 wks old mice were prepared. Intracellular expression of Ki67 in mTECp cells was measured to determine their proliferation. CD45^{-}EpCAM^{+} TECs were plotted. (**b**) Thymic stromal cells from 4 wks old *Ltbr*$^{fl/fl}$K14^{Cre} and *Ltbr*$^{fl/+}$K14^{Cre} mice were prepared. Intracellular expression of active caspase 3 in cTECs, mTECs and mTECp cells was stained to determine their apoptosis. Representative flow cytometer data is shown. The experiment has been repeated twice.

Figure S6. Epithelial LTβR does not control thymic emigration.

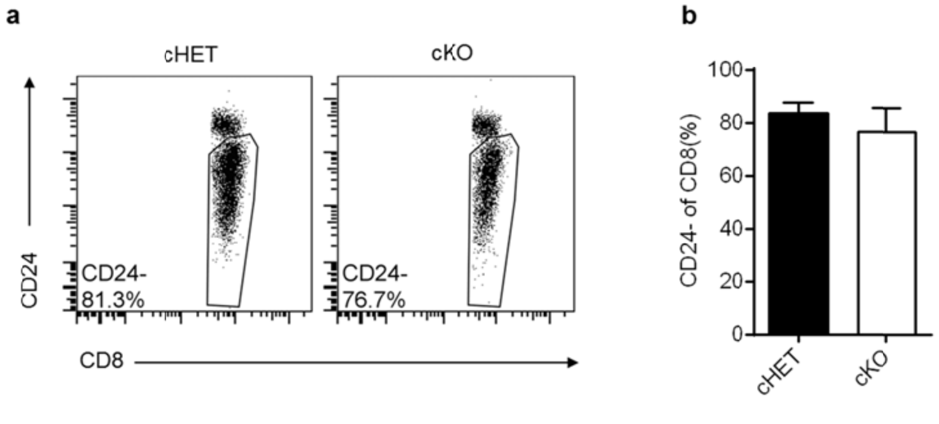

Figure S6. Epithelial LTβR does not control thymic emigration. Thymocytes from adult *Ltbr*$^{fl/fl}$K14^{Cre} and *Ltbr*$^{fl/+}$K14^{Cre} mice were prepared and then analyzed by flow cytometry. After gating CD8^{+}CD4^{-}T cell, the maturation of T cell was determined by CD24. Representative flow cytometer data (**a**) and the statistical data (**b**) are shown. Statistic data are mean ± SD for 5 mice in each group. An unpaired two-tailed Student's t-test is used. No significant difference was found between comparing groups. The experiment has been repeated twice.
